# Parasite induced mortality is context dependent in Atlantic salmon: insights from an individual-based model

**DOI:** 10.1038/s41598-019-53871-2

**Published:** 2019-11-22

**Authors:** Knut Wiik Vollset

**Affiliations:** NORCE Norwegian Research Centre, Laboratory for Freshwater ecology and Inland fisheries, Nygårdsporten 112, 5006 Bergen, Norway

**Keywords:** Conservation biology, Ecological epidemiology, Ecological modelling

## Abstract

An individual-based model was parameterized to explore the impact of a crustacean ectoparasite (sea louse, *Lepeophtheirus salmonis & Caligus spp*.) on migrating Atlantic salmon smolt. The model explores how environmental and intrinsic factors can modulate the effect of sea lice on survival, growth and maturation of Atlantic salmon at sea. Relative to other effects, the parasite infestation pressure from fish farms and the encounter process emerge as the most important parameters. Although small variations in parasite-induced mortality may be masked by variable environmental effects, episodes of high infestation pressure from fish farms should be observable in wild populations of Atlantic salmon if laboratory studies accurately reflect the physiological effects of sea lice. Increases in temperature in the model negatively influenced fish survival by affecting the development time of the parasite at a rate that was not compensated for by the growth of the host. Discharge from rivers was parameterized to increase migration speed and influenced parasite induced mortality by decreasing time spent in areas with increased infestation pressure. Initial size and growth of the host was inversely related to the impact of the parasite because of size-dependent parasite-induced mortality in the early phase of migration. Overall, the model illustrates how environmental factors modulate effects on the host population by impacting either the parasite load or the relative effect of the parasite. The results suggest that linking population-level effects to parasite infestation pressure across climatic and environmental gradients may be challenging without correctly accounting for these effects.

## Introduction

The role of parasites in regulating their host population and the role of parasite-induced mortality in population dynamics has been widely debated in parasitology and ecology^1,2^. Historically, it was thought that parasites had little effect on host survival except in rare occasions, and that individuals with high levels of parasites would ultimately succumb to factors such as predation or starvation rather than be killed by the parasites themselves. This was often attributed to the fact that fitness of the parasite is dependent on the host survival, and that the stable state equilibrium between host and parasites depends on the cost of the parasite to the host and the cost of developing different strategies to combat the parasite (e.g. immunocompetence or behavioral avoidance). In the wake of the pivotal papers by Anderson and May in 1978^1,2^, the role of parasites as a regulating factor is now recognized. However, whether mortality from parasites is additive (i.e. comes in addition to other sources of mortality) or compensatory (i.e. targets or culls weak individuals) is important to understand natural dynamics in parasite-host systems^3–5^.

In a natural system, the balance between hosts and parasites can vary between years, occasionally creating sporadic episodic outbreaks^6^. This balance can also be affected by humans through effects on climate^7^, introduction of invasive species that function as co-hosts^8^ or removal of predators that culls diseased individuals^9^. The role of disease interaction from fish farming with surrounding marine wildlife is an anthropogenic modification that has received scrutiny recently^10^. A large proportion of studies related to fish farming has focused on the salmon and salmon lice (or sea lice in general encompassing both *Lepeophtheirus salmonis & Caligus spp*., Krøyer, 1837) host-parasite complex^11–14^. The role of fish farms on the epidemiology of wild fish can be summed up into three major concerns: (1) amplification of parasite load decoupled from host density^15–17^; (2) temporal alteration of parasite production^18^; and (3) rapid human-induced evolution in resistance and virulence^19–21^. Alone or in combination, these may (or may not) impact populations of wild salmonids.

Studying the fate of treated and untreated Atlantic salmon (*Salmo salar*, Linnaeus, 1758) smolts released from hatcheries has been conducted since 1996 in Norway and Ireland (and in later years also in other countries). These studies demonstrated that parasites (most likely salmon lice) reduced survival^13,22^ and growth^23^ and increased the age of returning adult salmon^24^. However, the average effects were relatively small and strongly variable. The effect of sea lice on the physiology and survival of salmon smolts has been studied in a series of laboratory studies^25,26^. These studies have been used in risk assessments of sea lice-induced mortality of fish caught in the wild^10^ and are now the foundation of a management system in Norway that regulates biomass in fish farms by the risk of salmon smolt succumbing to sea lice emitted by fish farms^27^. The risk model uses threshold values of lice per gram fish to determine the risk posed to wild salmon. This necessitates either samples from wild fish or a model that can accurately predict the number of lice on fish and their size.

The above mentioned method of classifying risk assumes a size dependent effect of sea lice on salmon mortality rate, which decreases linearly with the hosts mass. Such size dependent parasite-induced mortality suggests that it is the smaller, slower growing and therefore more vulnerable salmon that will predominantly succumb to the parasite. If other mortality factors (such as predation) operate along the same axis, parasite-induced mortality can in theory be masked by the effect of other factors later in life. In addition, the existing risk assessment does not consider that the growth of both the host and the parasite are affected by the environment. For example, growth of both species is strongly temperature dependent^28^, and the differential impact on the development of lice and growth of salmon will potentially lead to differences in parasite induced effects along thermal gradients. Consequently, the link between marine survival of salmon and the infestation pressure of sea lice from fish farms most likely contains layers of complexity that are difficult to disentangle with field experiments that cannot manipulate all potential independent variables. Individual-based models (IBMs) can simulate scenarios and make predictions that avoid the limitations of field experimentation. Furthermore, salmon and sea louse are particularly suited for applying such models due to the availability of data on various aspects of the life history of the two species^14^. In this study, I present an individual-based model that explores how environmental and intrinsic factors can modulate the effect of sea louse on survival, growth and maturation of Atlantic salmon at sea.

## Material and Methods

This study follows the ODD (Overview, Design concepts, and Details) protocol proposed by Grimm, *et al*.^29^ and updated by Grimm, *et al*.^30^. The model was written in R (R Core Team 2018 v 2.15.3).

### Purpose

**Figure 1:**
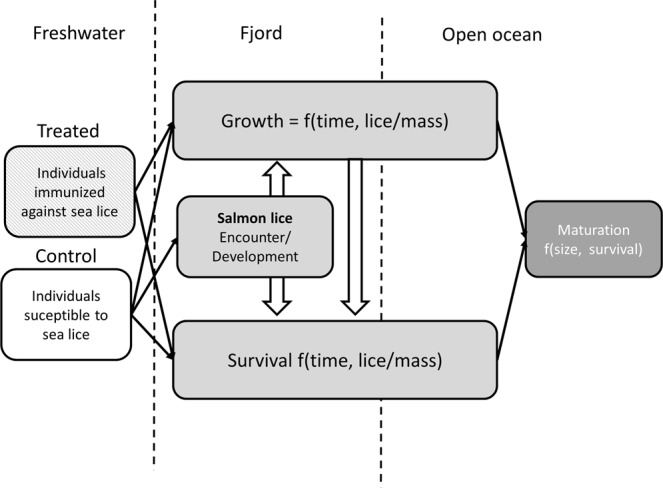
Simple conceptual diagram of model. Each box indicate a submodel and the parentheses indicate what the submodel is a function of. In addition the spatial configuration is indicated with dashed lines, i.e. either in freshwater, fjord, or open ocean. Black arrows indicate how the submodels feed into each other, while open large arrows indicate if the submodels is a function of each other.

The purpose of the model is to explore how louse encounters during the nearshore migration of Atlantic salmon smolts affects their marine survival, size, and maturation. The model was designed to simulate release group experiments in which groups of smolts are released simultaneously and half of the individuals are randomly assigned to a prophylactic treatment that alleviates the fish of parasites. Specifically, the model investigates how the relationship between infestation pressure of sea louse and marine survival are affected by environmental and intrinsic factors impacting the host and parasites. Finally, the model results are compared to the risk assessment suggested by Taranger, *et al*.^10^ and the role of marine growth and mortality in determining the fraction of overall mortality attributable to parasite effects is discussed (Fig. 1).

### Entities, state variables and scales

The entities of the models are individual salmon. The state variables include release date, age (in days from release), length (mm), weight (gram), migration speed (km × d^−1^), variation in sea growth (unitless), infection levels (including one state variable per sea louse life stage: copepodite, chalimus, preadult, adult), survival (yes/no), cumulative probability of survival and state of maturity (yes/no). Maturity is in the model defined based on the optimal age at maturation given the size and cumulative probability of survival as described in Hutchings and Jones^31^.

**Figure 2:**
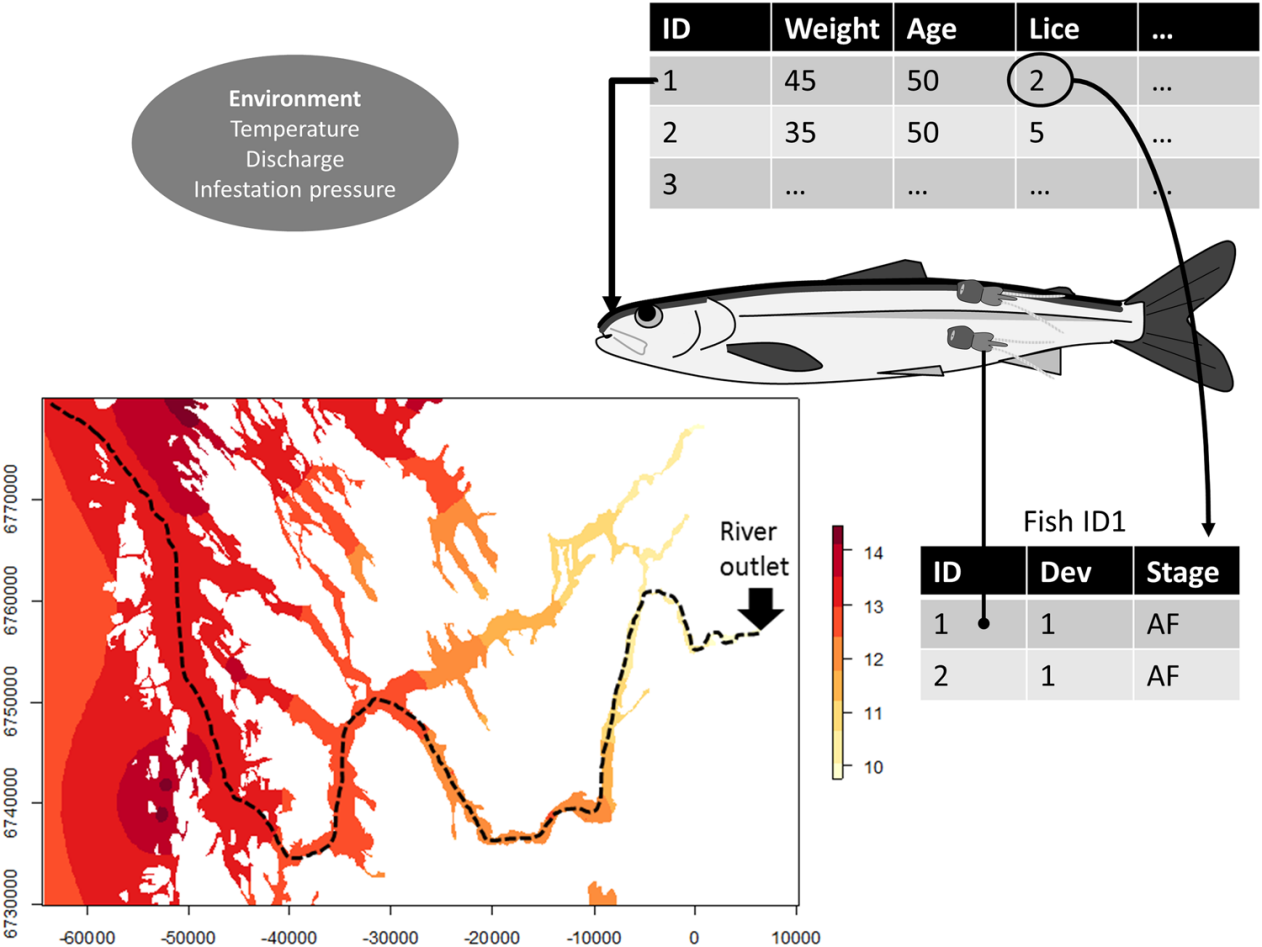
Illustration of model. Map of infestation pressure is taken for week 22 from 2016 for the migration route of the Vosso salmon, and illustrates how infestation pressure increases from the river outlet to the outer fjords. The stapled line is the likely migration route of the salmon smolt based on the prevailing currents in the region.

The model is spatially explicit during the nearshore migration (Fig. 2): fish are swimming from the release location to the edge of territorial waters (i.e. 12 nautical miles from land = 22.2 km) where they are subject to infestation pressure and encounter louse according to the relationship described in Kristoffersen *et al*. (2017). One time step represents one day and simulations elapse 150 days per release group. For simplicity, all individuals in the simulations are released the same day (15 May). This is a simplification of the out migration of salmon smolts, which generally migrate during a more prolonged period from May to July with a peak around mid May in this region. However, given that the infestation pressure does not have any temporal dynamics in this simplified model, this has little impact on the final outcome. The days remaining until maturation (265 days) were estimated in one time step, because preliminary runs of the model suggested that impacts of sea louse on growth and survival of survivors in this time interval was negligible.

### Process overview and scheduling

The simulated salmon were released in marine water. Each simulated smolt was assigned a random length from a normal distribution (µ = 135 mm, σ = 10) representative of wild smolt from the Vosso river in Norway^32^, and randomly assigned (50/50) to a treated or an untreated group. The migration speed of the fish was defined as a function of the body length and river discharge according to Vollset, *et al*.^33^. The number of days (i.e. time steps) within the nearshore environment (~100 km) was therefore dependent on the migration speed. Following each time step, the fish grew according to a temperature dependent growth function^28^. Simultaneously, the sea lice on the fish (which are tracked individually in their own IBM, Fig. 2) developed from copepodites to chalimi to preadults and finally to adults over the course of the days available for growth as a function of the temperature also experienced by its host. This means that sea lice are individual entities in their own model, but the sum of lice on each salmon can be viewed as a state variable in the salmon model. Temperature was set as a fixed number in each model run. Impacts of changing the temperature throughout the season was explored, but had relatively small impact on the outcome of the model. The likelihood of host survival was a function of host length and number of preadult and adult lice per fish gram. At the end of the simulation (415 days), the model evaluated whether the fish was mature or not based on a simple Euler-Lotka equation described in Hutchings and Jones^31^.

### Design concept

#### Emergence

Survival (and consequently estimated mortality attributed to sea lice – i.e. attributable fraction) and maturation emerged as a result of the combined stochastic processes of louse encounter, marine growth and mortality.

#### Adaptation

Fish mature according to cumulative mortality and growth by the end of the year.

#### Objectives

The individuals have no predefined objectives; their behavior in the simulation arises entirely from initial values and stochasticity.

#### Learning

There is no learning involved

#### Prediction

The decision to mature is based on a predicted growth and survival the following years.

#### Sensing

The individual fish can sense temperature and encounter louse according to the infestation pressure from fish farms defined in a spatial raster. The temperature affects the fish length and the development of louse on the fish. In addition, discharge from the river impacts their migration speed through the fjord system and therefore the exposure to lice.

#### Interaction

There are no interactions between individuals.

#### Stochasticity

Initial length distribution (which is also converted to mass) is drawn from a normal distribution rendering different growth trajectories based on the temperature dependent growth model. In addition, the louse encounter and survival is stochastic in the sense that it is drawn from random negative-binomial and binomial probability functions, respectively.

#### Collectives

Individuals are assigned randomly to either a treated or untreated fish within each release group.

#### Observations

The main observations from the model are mass, survival and maturation by the end of the year, and the mortality attributed to sea lice based on the relative survival between the treated and untreated individuals. This was calculated by first running the model for the first 150 days with daily updates, and then the remaining time until river return, which was defined as July 5, in one time step (i.e. 265 days). In some instances, the mass and lice encounters of all individuals (surviving and not surviving) during the first two months was logged to illustrate how development of the lice, initial mass, growth and infestation pressure affects the host probability of mortality during the initial phase of migration.

### Initialization

Initially, individuals were assigned a release date, release length (total length*, L*_*T*_) and a treatment group (treated / not-treated). Mass was derived from a linear mass-length relationship fitted to data from the Vosso system

1 $$M=0.0273+\frac{{L}_{T}}{10}\times 2.5228$$

where M is the individual mass and *L*_*T*_ is length drawn from a normal distribution (µ = 135 mm, σ = 10).

### Input data

The model inputs are discharge during migration, infestation pressure from fish farms and temperature.

### Submodels

#### Growth

Specific growth rate of Atlantic salmon (sgr) is temperature (T) dependent according to Handeland, *et al*.^34^

2 $$sgr=-\,0.000117+0.011395T+0.018436{T}^{2}-0.000977{T}^{3}$$

To increase and decrease the growth rate the second parameter was multiplied by a factor of 2 and 0.2. This changed the growth rate by approximately 10% up or down throughout the thermal range.

The effect of sea lice on the specific growth rate is not known, but Susdorf, *et al*.^35^ suggested that a 10% reduction in growth may be valid. Also, Skilbrei, *et al*.^23^ showed that the size of returning after one winter at sea (1SW) salmon was ~100 gram larger when a fish received treatment against sea lice as smolt. Therefore, a simple linear relationship between sea lice infestations and reduction in growth was applied as follows:

3 $$reduction=1+LPG\times -\,0.33$$

in which LPG is mobile lice (preadult and adult) per gram fish. This suggests that there will be 10% reduction in sgr when an individual has 0.3 lice per gram. This is the same level of reduction in growth used by Susdorf, *et al*.^35^. There is however, little data to quantify the exact impact of sea lice on growth. The resulting *sgr* for the impacted fish will consequently be *reduction* × *sgr*.

#### Migration

To model progression rate of salmon, the model from Vollset, *et al*.^33^ was applied, which estimated daily progression rate based on release and recapture of coded-wired-tagged smolt in and outside the Osterfjord in Western Norway. A simplified version of their linear model was applied in which the migration speed (in kilometers per day) is solely a function of length of the fish and discharge from the Vosso river.

4 $$v=-\,1.07+0.082\times {L}_{T}+0.017\times D$$

in which *v* is the daily migration rate of smolts in *km* × *d*^−1^, *L*_*T*_ is the total length of the fish in mm, and *D* is the discharge from the river Vosso measured as ${m}^{3}{s}^{-1}$. This was used to calculate the number of days spent in the nearshore environment by rounding *v* divided by migration distance (in km) to the nearest whole number. To increase or decrease the migration speed, the intercept was multiplied by a factor of 3 and 0.5. This changed the swimming speed by approximately 10% up or down throughout its range.

#### Sea lice encounter

Simulated salmon smolts encountered parasites according to the parameterized model by Kristoffersen, *et al*.^36^, which linked infestation pressure from fish farms to lice attached to smolts in sentinel cages during a period of 20–25 days. The infestation pressure (IP) is based on the estimated total external infestation pressure from fish farms on the Norwegian coast. The estimated IP from the model is a unitless value (on a log scale) and normally varies between 5–17. The expected number of lice encountered by a fish during one day is calculated based on the following equation

5 $${\rm{TP}}={{\rm{e}}}^{-14.063+0.843IP}$$

were TP is defined as the transmission pressure, and was used draw a random number from a negative binomial distribution, parameterized such that mean(x) = µ and var(X) = µ + µ^2^/theta, with µ = TP and dispersion parameter theta = 2.733 (as quantified in Kristoffersen, *et al*.^36^). By varying theta from 1 to 5, it was possible evaluate to what degree patchiness impacted the final outcome of the model.

To make comparable model runs, the infestation pressure from week 22 in 2016 from the migration route of the Vosso salmon was extracted (Fig. 2). The data extracted were provided by the veterinary institute, and can be viewed online here: http://apps.vetinst.no/lusekart. This step was taken because the migration speed model is validated from this region^33^, and detailed knowledge about salmon migration time in this region is known from trap netting^33^. High and low infestation pressures were modelled by multiplying the infestation pressure extracted as explained above (i.e. baseline infestation pressure) by a factor of 1.2 or 0.8, yielding values comparable to high and low infestation pressure estimated from Kristoffersen, *et al*.^36^. Histograms resulting from modelled encounters with lice for the three infestation pressures are provided in the appendix. These values are comparable to the lice numbers observed on trawled post-smolts during sea lice surveys along the Norwegian coast^25^.

#### Sea lice development

Sea lice are modelled individually and each individual louse has a temperature dependent development. The developmental time from attached copepodids through chalimus to mobile (preadult and adult) developmental stages, was taken from Stien, *et al*.^37^ and modified as explained in Groner *et al*. (2013). The equation used to model development is the modified Belahrádek equation, and has been used in multiple publications on salmon lice:

6 $${\tau }_{ij}(T)={(\frac{{\beta }_{1ij}}{(T-10+{\beta }_{1ij}{\beta }_{2ij})})}^{2}$$

where τ_*ij*_ is the minimum required development time for salmon lice *i* in stage *j* at temperature *T*. β_1_ is a shape parameter, and ${{\rm{\beta }}}_{2}^{-2}$ is the average τ at 10 °C. As in Groner *et al*. (2013), a stage‐specific constant, ν_*j*_, was added to this temperature‐dependent estimate, to represent additional time beyond the minimum development time. Lice development from eggs to chalimus attached copepodit are integrated in the infestation pressure estimate as explained in the previous methods. Thus, parameters are only needed for the developmental time for chalimus and preadult (Table 1). In addition, each louse had a daily probability of surviving (δ) based on the parameters from Stien *et al*. (2005). The mortality of adult lice on pelagic swimming post-smolts is assumed to be low (~0%), based on the assumption that the mortality seen in laboratory studies is mainly due to contact with physical barriers not present in the wild. Either way, the mortality parameter of adult lice did not have a large impact on the overall outcome of the model.

**Table 1:** Parameters of lice development for chalimus and preadult stages as described in Eq. 6.

Life stage	β1	β1	v
Chalimus	74.7	0.236	0.85
Preadult*	64.7	0.177	0.34

#### Survival

Daily natural mortality of salmon (day^−1^) was quantified using the relationship by Peterson and Wroblewski^38^

7 $$Mp=5.3\times {10}^{-3}{({10}^{-3}DW)}^{-0.25}$$

in which Mp is the daily natural mortality and DW is the individual dry weight of the fish. DW is assumed to be 25% of the wet weight as logged in the model (M). In an earlier version of the model, the mortality function used in Piou and Prévost^39^ was applied and yielded similar results. The alternative version from McGurk^40^ was also tested, but was clearly not parametrized for the size range of salmon smolts and was therefore abandoned.

**Figure 3:**
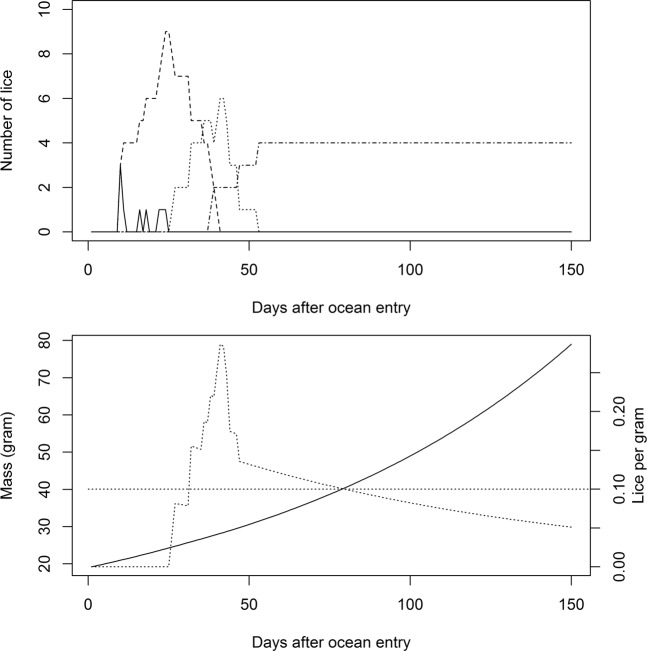
Upper panel; A random example of the development of lice on a salmon post smolt. Copepodits (solid lines), chalimus (dashed line), preadult (dotted line), and adult (dashed and dotted lines) number of lice appears subsequently as the parasite attaches and develops according to temperature. Lower panel: increase in mass according to the temperature dependent growth model (solid line) and the resulting mobile lice per gram fish (dotted line). 0.1 lice per gram, which has been defined as the threshold for effects of salmon lice on post-smolts, is indicated with a straight dotted line.

Lice dependent mortality was based on the threshold limits presented in Taranger, *et al*.^10^, fitted to a logistic regression (see appendix for details), rendering the following equation:

8 $$log[\frac{p}{1-p}]=4.721-16.51\times LPG$$

where p is the probability of survival and LPG is lice per gram fish. This was used to calculate the likelihood of surviving parasite infections (p) if LPG was >0, and set to 1 if LPG = 0. In the model p is interpreted as a daily survival rate. In reality, the values presented in Taranger, *et al*.^10^ are not daily rates, but rather overall risk of death during the smolt migration such that p = 1-m, where m is the probability of surviving given an LPG. Finstad, *et al*.^25^ demonstrated that mortality increased after the lice developed to preadult stages. Lice per gram is therefore number of mobile lice divided by the mass of the fish. Lice per gram and consequently mortality rate (m) therefore increases after lice develop into preadults (Fig. 3). No one has previously attempted to calculate the daily mortality rate according to sea lice (Ml). Attempts were made to extract data from publications to calculate the daily mortality rates, but the details in the publication were scarce for such estimates. For simplicity, I divided the estimate of m by five assuming that the mortality takes place during a 5 day period such that Ml = m/5. During a standard run with baseline values this would estimate an average attributable fraction of ~50% for individuals with 4–6 lice, which is comparable to the risk model suggested by Taranger, *et al*.^10^, and the values used by Kristoffersen, *et al*.^36^. The total daily probability of survival (Sp) following Eqs. 7 and 8 is as follows

9 $$Sp={e}^{-Mp}\times (1-Ml)$$

#### Maturation

Maturation is calculated after the model run based on the Euler-Lotka equation parametrized by Hutchings and Jones^31^

10 $$1=\mathop{\sum }\limits_{x=\alpha }^{x=\omega }\,{l}_{x}{m}_{x}{e}^{-rx}$$

where, for ages *x* between maturity *α* and death $\,\omega$, *l*_*x*_ represents the survival probability from birth to the beginning of the spawning season at age *x*, and *m*_*x*_ represents the number of zygotes produced by an individual spawning at age *x*, while *r* represents the population growth rate. By turning the equation it is therefore possible to calculate *r* for different maturation strategies, i.e. maturing after one year or multiple years at sea (1SW versus MSW). I use the parameters extracted for the Vosso salmon for this study. This entails calculating ${l}_{x}$ as the product of the survival from egg to smolt set to 0.15% and the survival from smolt to 1SW (which is calculated as the product of daily mortality in Eqs 8, 9 and 10), and calculating *m*_*x*_ (i.e. fecundity) as a function of the length of the fish based on the equation

11 $$F=0.4667+{L}_{T}\times 2.2018$$

where F is fecundity and *L*_*T*_ is total length. The estimate of *r* maturing after 1 year at sea (*r1*) is then compared to the *r* for an individual maturing after to year at sea (*r2*). To be able to calculate *r2*, the growth and survival the second year at sea was set to fixed as 40% increase and 55% survival, which is comparable to the values used in Hutchings and Jones^31^. An individual thus matures as 1SW if r1 > r2.

### Explanation of validation

Submodel validation was accomplished by a combination of face validation, rationalism and extreme condition tests^41^. This involved running sub models across a range of values and evaluating if the outcome was within a biologically reasonable range using graphical outputs. For example, the output of the growth model was evaluated against the observed size range of salmon observed according to scale analysis of salmon from the Vosso river^42^, whereas the lice encounter model was evaluated by comparing the lice encountered by salmon in the model with the number of lice observed during surveillance program along the Norwegian coast. All submodel outputs that fell within observed ranges of empirical data were deemed valid. For the final outcome (i.e. the emerging properties survival, growth and maturation), a sensitivity analysis was conducted (see 10).

### Comparison with risk evaluation of sea lice

The current Norwegian risk evaluation of sea lice on salmon uses a mortality rate based on lice per gram salmon smolt, which corresponds to a mortality of 0% if there are <2 lice per fish, 20% if 2–4 lice, 50% if 4–5 and 100% if >6 lice for a 20 gram salmon smolt^10^. These are the same threshold values used in Kristoffersen, *et al*.^36^. To explore how the IBM compares with the current risk assessment, I used the estimated mortality calculated from the number of lice encountered in the infestation module of the model combined with the risk assessment model by Taranger, *et al*.^10^, and compared it with the estimated mortality from the IBM.

### Sensitivity analysis

A simple one-at-a-time (OAT) sensitivity analysis was conducted with the outcome of interest being mortality attributed to sea lice (Attributable fraction, AF), untreated group survival, marine growth, and maturation. All model parameters, description and their range is given in Table 1. The variables that were tested and the maximum and minimum values are given in Table 2.

**Table 2:** Values used in OAT sensitivity analysis.

Parameter	Parameter				
	Parameter name	Values			units
		Min	Baseline	Max	
Temperature	T	6	10	16	°C
Average initial length	µ	110	140	200	mm
Patchiness^a^	theta	1	2.733	5	
Growth function	See appendix	See appendix			
Swimming function	See appendix	See appendix			
Average discharge^b^	D	50	150	300	m^3^s^−1^
Salmon lice induced mortality	See appendix	See appendix			
Encounter rate function	See appendix	See appendix			
Mortality equation	See appendix	See appendix			

Parameter name corresponds to the description given in Table 1. “Min” signifies minimum values, “Max” signifies maximum values, while baseline indicates the values for the parameter when other variables were changed accordingly. ^a^Used in Eq. 5, ^b^used in Eq. 4.

## Results

### Examples of development of lice on fish

Figure 3 illustrates a random sample of a salmon post-smolt encountering 9 lice during the early marine migration and the temporal development of lice, the mass of the fish and the resulting lice per gram. The highest value of lice per gram is around day 45, which is almost 1.5 months after the post-smolt has left the river(Fig. 3). Temperature impacted the timing of peak virulence of sea lice on migrating post-smolt for a period of 1.5 months, which is much longer than the short period of time that is spent migrating in the coastal environment. Thus, measurements of temperature in the coastal environment will not be sufficient to understand how temperature impacts lice development on wild fish.

### Sensitivity analysis

**Figure 4:**
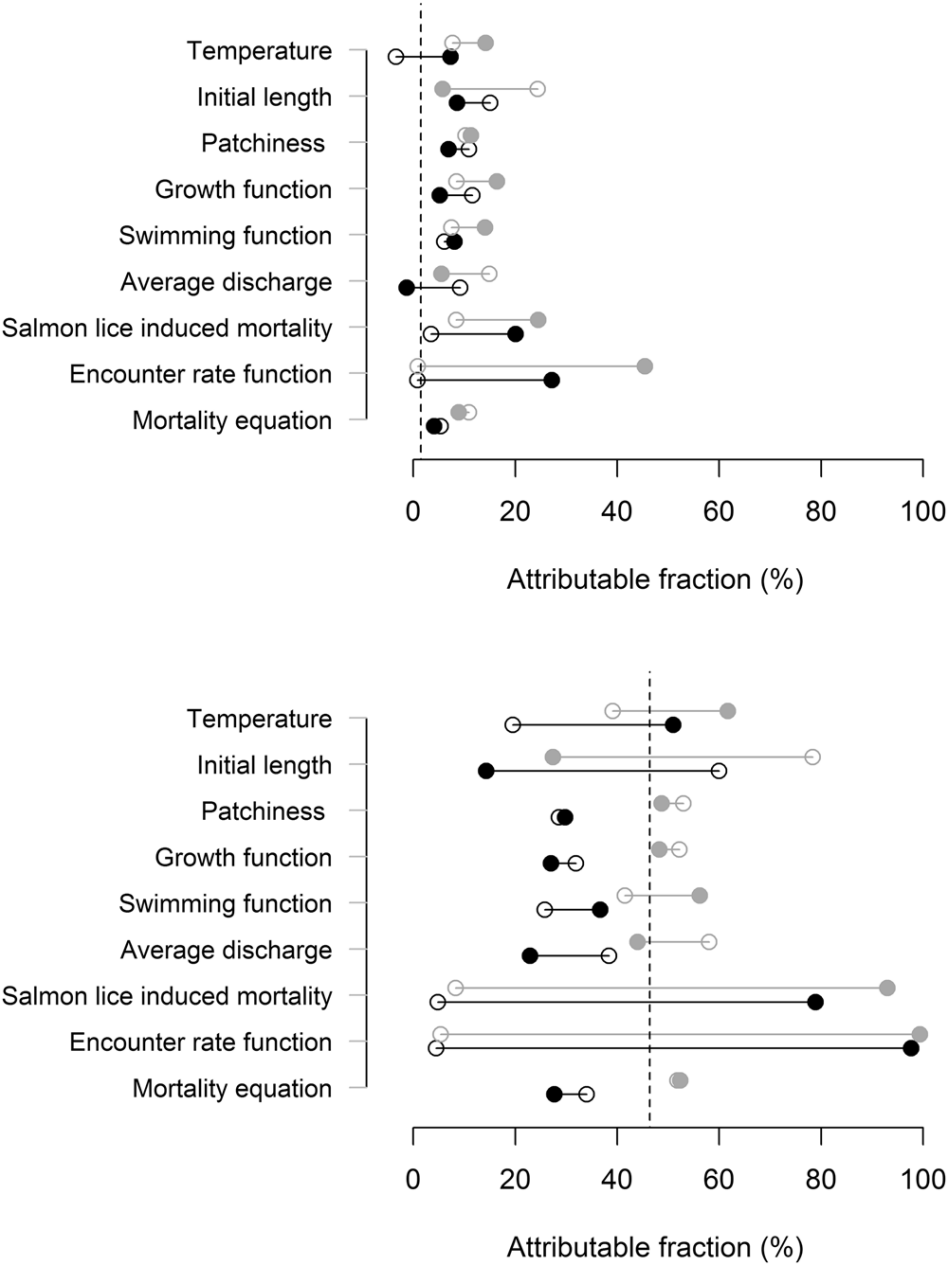
Values of attributable fraction of sea lice from OAT sensitivity analysis from model including (grey) and excluding (black) an effect of sea lice on growth. Open circles indicate model using minimum value, while solid circles indicate model using maximum values The names of the parameters are described in Table 3. The values are also given in Tables 3 and 4. Upper panel are models using baseline infestation pressure, while lower panel is the model using high infestation pressure. The dashed lines indicate the resulting mortality estimates using the risk model from Taranger et al. (2015).

Resulting attributable fraction, overall marine survival, final weight and proportion maturing as 1SW are given in Tables 1 and 2 for the selected parameters. In Table 3, the model is parameterized with standard baseline values. In Table 4, the model is parameterized with an assumed effect of lice on weight as explained in section “Growth” (Eq. 3). Estimates are given for baseline values of infestation levels (upper tables), in addition to high infestation levels (lower tables) to be able to evaluate the effect of the different parameters during episodes of high lice loads on the post-smolt (see section Sea lice encounter for a description of high infestation pressure). The resulting AF is also plotted in Fig. 4. The results from the sensitivity analysis suggest that the most sensitive parameters were the functions linking parasite load to mortality and the encounter rate function, indicating that the modelled encounter with lice and resulting mortality were important parameters. Indeed, this result has been demonstrated before by Kristoffersen, *et al*.^36^, which showed that changing the mortality function and the link between infestation pressure and modelled lice had a large effect on the estimated mortality. Other important parameters were temperature, initial size of the fish, the growth and swimming functions and the discharge. Patchiness had a small effect on the outcome of the model. The natural mortality function (size-selective mortality) had a strong effect on the overall survival and maturation of the individuals, but had seemingly no measurable effects on the effect of parasites on survival of salmon (see appendix for the three parameterizations of size selective mortality). Finally, comparing the two tables it is evident that a parameterized effect of lice on growth generally increases the impact of lice on survival and maturation.

**Table 3:** Sensitivity analysis in which the model is parameterized with no effect of sea lice on growth rate on their salmon host.

Parameter	Minimum				Maximum			
	AF (%)	SC (%)	FW	PM	AF (%)	SC	FW	PM
**Baseline infestation pressure/no Growth effect**								
Temperature	−3.4	5.6	601	0.07	7.3	6.4	1265.0	0.60
Initial length	15.1	3.6	629	0.05	8.6	11.5	3093.3	0.99
Patchiness	10.9	6.4	1201	0.64	6.9	6.9	1200.2	0.61
Growth function	11.6	6.2	1048	0.53	5.2	7.0	1432.6	0.72
Swimming function	6.1	6.7	1194	0.64	8.1	6.4	1207.1	0.61
Average discharge	9.2	6.4	1194	0.57	−1.3	6.8	1224.7	0.70
Salmon lice induced mortality	3.5	6.7	1178	0.70	20.1	5.6	1212.2	0.70
Encounter rate function	0.8	6.9	1209	0.76	27.2	5.1	1241.0	0.18
Mortality equation	5.39	94.61	1140	1.00	4.09	1.1	1335	0.03
**High infestation pressure / no Growth effect**								
Temperature	19.5	4.7	623	0.06	51.0	3.4	1339	0.12
Initial length	60.0	1.6	681	0.01	14.3	10.7	3108	0.87
Patchiness	28.6	5.0	1234	0.18	29.8	4.9	1217	0.14
Growth function	31.9	4.6	1111	0.14	27.0	5.2	1484	0.19
Swimming function	25.8	5.3	1238	0.28	36.7	4.5	1234	0.14
Average discharge	38.4	4.2	1252	0.09	22.9	5.3	1226	0.31
Salmon lice induced mortality	4.8	6.6	1213	0.63	78.9	1.5	1319	0.43
Encounter rate function	4.5	6.8	1206	0.59	97.7	0.2	1569	0.00
Mortality equation	34.0	66.0	1184	0.84	27.7	0.9	1352	0.00

The values for the parameters at minimum and maximum values are given in Table 2. AF signifies attributable fraction, SC survival in untreated group, FW final weight and PM proportion of population matured after one winter at sea.

**Table 4:** Sensitivity analysis were the model is parameterized with an effect of sea lice on growth rate on their salmon host as described in Eq. 2.

Parameter	Minimum				Maximum			
	AF (%)	SC (%)	FW	PM	AF (%)	SC	FW	PM
Temperature	7.7	5.1	617	0.07	14.18	6.08	1274	0.61
**Baseline infestation pressure/Growth effect**								
Initial length	24.4	3.2	625	0.05	5.78	11.655	3078	0.95
Patchiness	10.3	6.4	1206	0.59	11.33	6.18	1166	0.53
Growth function	8.5	5.9	1082	0.49	16.43	6.205	1432	0.66
Swimming function	7.5	6.3	1202	0.64	14.10	6.185	1190	0.56
Average discharge	14.9	6.2	1181	0.51	5.56	6.875	1203	0.65
Surv per lice per gram	8.4	6.5	1199	0.69	24.54	5.12	1203	0.70
Encounter rate function	0.8	7.0	1205	0.71	45.45	3.865	1229	0.09
Mortality equation	10.9	89.1	1137	0.97	8.9	1.1	1369	0.029
**High infestation pressure/Growth effect**								
Temperature	39.1	3.4	652	0.02	61.7	2.71	1372	0.07
Initial length	78.4	0.8	701	0.01	27.4	9.24	3093	0.63
Patchiness	53.0	3.6	1249	0.10	48.7	3.61	1266	0.08
Growth function	52.2	3.2	1119	0.07	48.3	3.7	1458	0.06
Swimming function	41.5	4.1	1245	0.15	56.2	3.175	1279	0.07
Average discharge	58.1	2.8	1255	0.03	44.0	3.92	1220	0.17
Surv per lice per gram	8.3	6.5	1171	0.50	93.0	0.48	1296	0.43
Encounter rate function	5.4	6.4	1225	0.57	99.4	0.04	1617	0.00
Mortality equation	51.8	48.2	1195	0.61	52.4	0.59	1267	0.00

The values for the parameters at minimum and maximum values are given in Table 2. AF signifies attributable fraction, SC survival in untreated group, FW final weight and PM proportion of population matured after one winter at sea.

### Temperature

**Figure 5:**
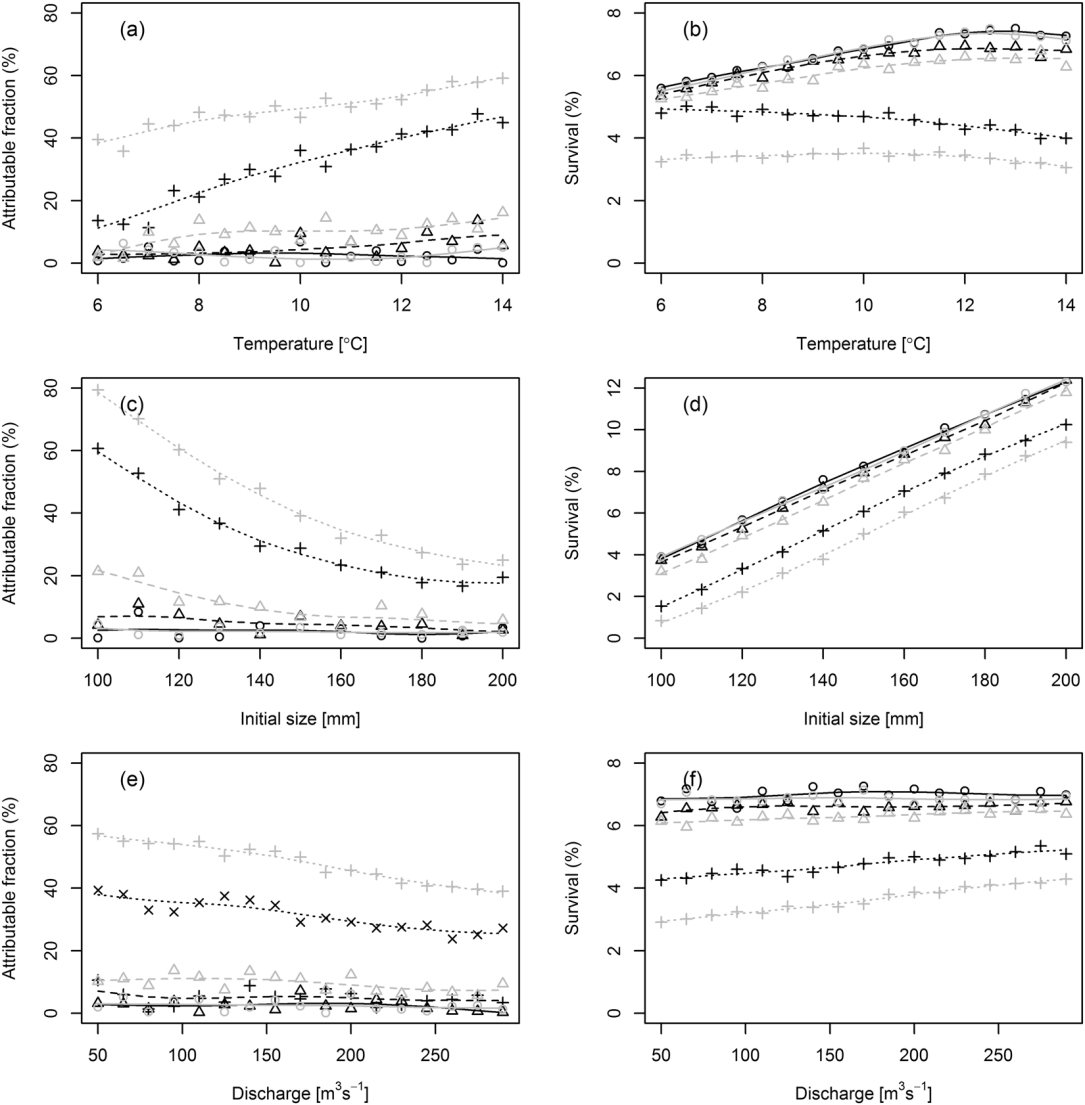
Attributable fraction of population succumbing to parasitic sea lice and overall survival from the individual-based model parameterized with baseline infestation pressure levels (triangles and dashed line), and high infestation pressure (crosses and dotted line). Each value are plotted against temperature (**a,b**), initial size (**c,d**), and discharge (**e,f**). Grey and black lines indicate models parameterized with or without an effect of sea lice on the growth rate of the salmon host.

**Figure 6:**
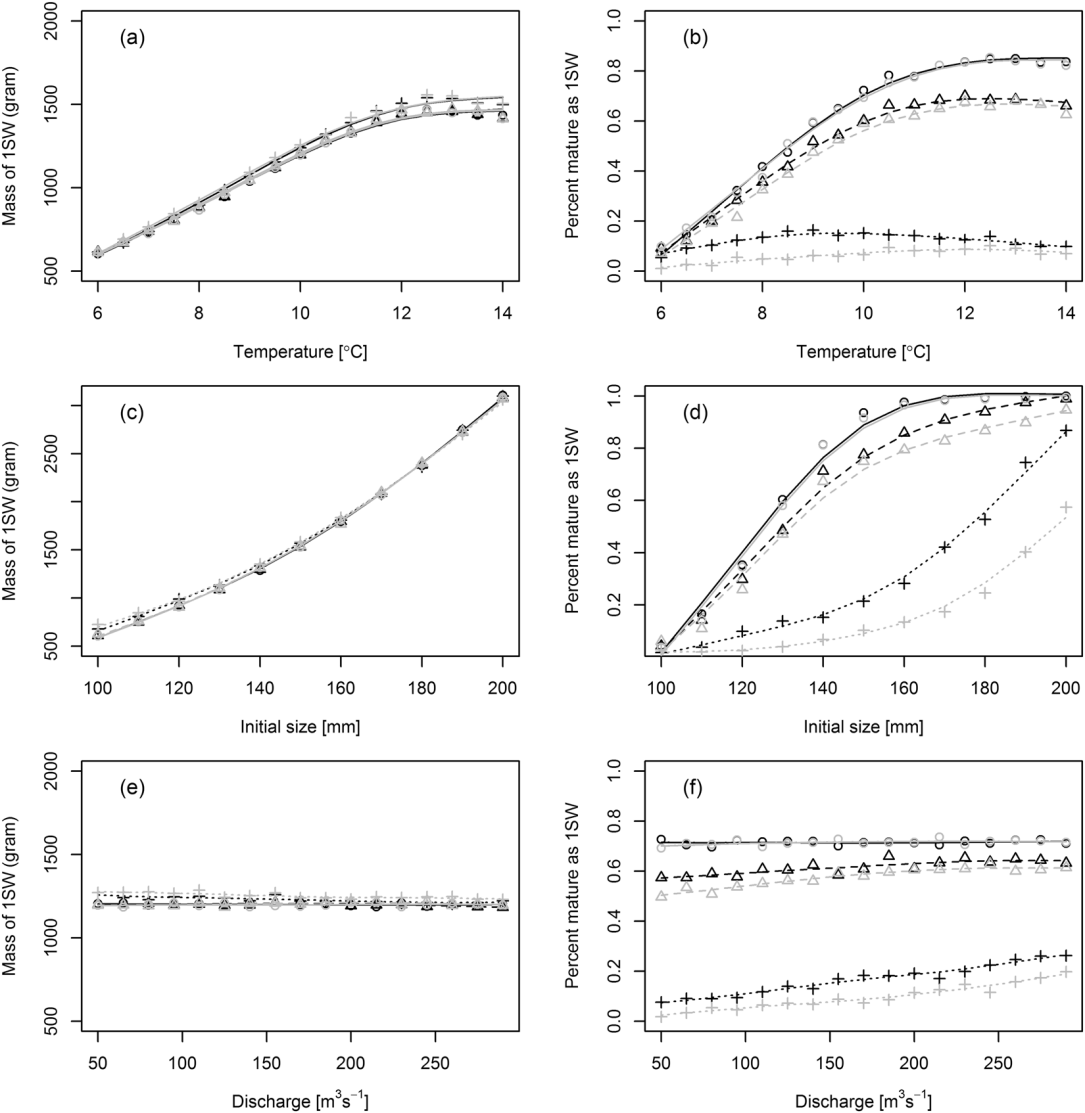
Size of salmon after 1 year at sea (415 days) and proportion maturating after 1 year at sea from the individual-based model parameterized with low infestation pressure levels (circles and solid line),with baseline infestation pressure levels (triangles and dashed line), and high infestation pressure (crosses and dotted line). Each value are plotted against temperature (**a,b**), initial size (**c,d**), and discharge (**e,f**). Grey and black lines indicate models parameterized with or without an effect of sea lice on the growth rate of the salmon host.

The effect of parasites increase with temperature (Fig. 5a). This is a result of the temperature dependent growth and size dependent mortality. The untreated group impacted by parasites was also impacted by temperature through the temperature dependent developmental time of the parasite. This yielded an increased impact of lice at high temperature and high lice infestation levels. The faster growth rate of salmon smolts cannot compensate for the decreased developmental time for the parasite. This is also illustrated in the overall survival of the untreated group, which shows that the survival increases with temperature in groups experiencing low or baseline infestation levels, but decreases with temperature when the model is parameterized with a high infestation pressure (Fig. 5b). Parameterizing an effect of lice on growth increased the attributable fraction due to parasite mortality, but decreased the effect of temperature (i.e. decreased the slope between temperature and AF). The ultimate size of the returning salmon increased with temperature, but there were almost no observable differences between groups from low or high infestation pressure in size of surviving individuals (Fig. 6a). Proportion maturing as 1SW increased with temperature in low and baseline infestation levels. For high infestation pressure, proportion of 1SW did not increase, but was low throughout the temperature range (Fig. 6b). In addition, parameterizing an effect of lice on growth decreased the proportion maturing as 1SW slightly.

### Size of released fish

The effect of parasites decreased with increasing size of fish (Fig. 5c). This was particularly evident in groups with high infestation pressure, but was only evident in baseline infestation pressure settings when the model included an effect of lice on growth. This effect was relatively small in a natural range of wild salmonids (120–150 mm) but was evidently large when compared with sizes that are sometimes observed among hatchery reared fish (200 mm). Similar to temperature, initial size was important for growth (Fig. 5, mid left) and age at maturity (Fig. 6d).

### Discharge and swimming speed

Increased discharge yielded faster progression speed through the fjords, reducing the days in areas with increased infestation pressure from fish farms. The effect was relatively small compared to the effects of temperature and initial size (Fig. 5e), and was very similar to increasing the swimming speed of the fish. However, the effect should be observable if studies are conducted in extreme conditions of spring floods versus periods of very low discharge. Discharge had very little observable effect on growth (Fig. 6,e) and maturation (Fig. 6f).

### Growth

Increasing growth rate of the salmon smolt (for example in a scenario were prey were abundant) had an opposite effect to increasing growth of salmon smolts by increasing temperature. This occurs because increased growth allows the fish to outgrow the negative impact of sea lice. This was the same effects observed when increasing initial size. This was more important when infestation pressure was low compared to when the infestation pressure was high, because the relative effect of size was more important when lice loads was low.

### Comparison model to risk model

The resulting risk estimated mortality estimates from the low, baseline and high infestation pressure using the model from Kristoffersen, *et al*.^36^ was 0.012%, 1.5% and 46.5%. Results from the OAT model suggests that the risk model generally underestimates the mortality compared to the IBM model for baseline values of infestation pressure (Fig. 4 – dashed lines). For high infestation pressure, the risk estimates could over- or underestimate the mortality depending on the values of the other factors.

## Discussion

Manipulative studies have demonstrated that parasites such as sea lice affect marine survival^13,22,23,43^. However, linking infestation pressure from fish farms or parasite abundance observed on fish *in situ* to measurable effects of the parasites on fish populations has been challenging. For example, in an attempt to model the impact of sea lice from fish farms on wild fish, Vollset, *et al*.^13^ used an infestation pressure model described in Jansen, *et al*.^44^, but could not account for heterogeneity in the effect of antiparasitic treatment on release groups of salmon. Instead they found that the heterogeneity in treatment was found to be strongly linked to the baseline survival of the release group. In other words, they found a positive effect of antiparasitic treatment on survival of salmon when marine survival was relatively low, but a smaller effect when marine survival was high. According to the authors the effect of the parasite is therefore likely modulated by other factors operating on the survival of salmon. Accounting for all possible factors in the wild that may modulate parasite induced mortality is extremely difficult; simulation studies assist in bridging these knowledge gaps and bring context to observations of fish in the wild. The results from the model presented in this study demonstrate that several environmental factors can impact the effect of parasites populations of salmon and can be summarized into three main novel findings: (1) temperature mediated growth indicates that effects of sea lice should be stronger with higher marine water temperature. This can have implications both for the effect of parasite-induced mortality as the season progress, as well as the impacts of different populations along latitudinal gradients and in the face of climate change; (2) initial size or growth can modulate the effect of sea lice-induced mortality; and (3) conditions that decrease the time spent in the nearshore areas (such as high river discharge or swimming speed) will reduce impacts of sea lice on salmon.

The model suggested that temperature can have an impact on the severity of parasite-induced mortality. This impact can be mainly attributed to the fact that the host (salmon) and the parasite (sea lice) respond very differently to temperature^28,37^. Compared to the shorter-lived sea lice^37^ salmon develop at a much slower rate^28^. This means that at an equal sea lice infestation, lice reach the mobile stage, which is known to potentially do damage to the host^25^, at a much larger parasite:host size ratio than at colder temperatures. Warming sea temperatures are therefore predicted to shift the balance in the parasite-host complex of sea lice and salmonids. If this prediction holds, it should also be expected that sea lice is more harmful for salmon populations at the southern boundaries of their distribution. Another important aspect is that parasite distribution among their hosts is usually strongly skewed such that a few individuals host many parasites, whereas a large proportion of potential hosts has small levels of infestation. It is therefore important to understand the physiological effects of warming waters on parasite-host interactions when abundance of parasites are low in order to make ecologically accurate predictions which are valid on a population level.

Size at release was also an important parameter in the model, indicating that the effect of lice increase curvilinearly with decreasing size. In tagging and cultivation programs, size is often used to compensate for the lower returns of cultivated fish compared to wild fish^45^. Consequently, usually large fish are used in studies releasing treated and untreated salmon smolt^13^. The model suggests that future trials should attempt to specifically test the interactive effect of release size and parasite-induced mortality, something that is revealed to be important, but lacking in empirical studies. This effect can also be relevant for the understanding of the impacts of wild fish as size-at-sea-entry may vary between populations, latitude, and among years.

Similar to initial size, decreased growth rate was related to increased parasite-induced mortality. In this model, growth was purely modulated by temperature. In reality, however, growth in wild salmonids is strongly impacted by their encounter with suitable prey. However, as of now no model exists that can correctly model the post-smolt growth of Atlantic salmon, although analysis of scales has given us insights into annual and seasonal trends in growth and how it may vary with climate^46^. The general trend in the North Atlantic has been declining growth and survival in southern populations, which has been attributed to large scale ecosystem regime shifts^47^. In relation to parasite-induced mortality, variable marine growth, coupled to poor survival, may be the underlying mechanism behind the strong correlation observed between survival and parasite-induced mortality^13^. From a salmonid ecology point of view, we are still scraping the surface of the mechanisms that govern the marine survival and growth of salmon.

Discharge and migration length both had an intermediate effect on the impact of sea lice. This emerges in the model because of the longer exposure time due to lowered progression rates through the fjord. If fish migrate during peak flow, as shown in some acoustic telemetry studies^48^, it will strongly reduce the effect of sea lice. Peak flows are projected to be more contracted with climate change because of flashier river dynamics associated with less snowfall and more rapid melting. Mismatched timing between spring freshets and productive ocean conditions could imperil smolt runs as they either enter the ocean early when conditions are poor or late when lice pose a greater threat. Discharge may, in addition to affecting the progression rate of smolts through estuaries and fjords, affect the salinity that the smolt encounter during migration. Sea lice are known to avoid low salinity water^49^, and this effect may increase the importance of river discharge during smolt migration. Although this model does not account for this affect, the relationships among river discharge, estuary salinity, sea lice abundance, and smolt survival should be explored in future studies.

The encounter rate function had a relatively strong effect on the simulation outcome. This was also demonstrated in Kristoffersen, *et al*.^36^. Similarly, the effect of the function that relates lice per gram fish to mortality was important. Together with the infestation pressure, these variables seemed to be the most sensitive parameters in the model, reinforcing that efforts must foremost minimize uncertainty around these parameters. Various institutions in Norway and internationally have attempted to model the distribution of sea lice larvae using hydrodynamic models to be able to predict encounter rates with sea lice in space and time as post smolts embark on their marine migration^50–52^. Modelling encounter rates are key ecological concepts, and various model frameworks^53,54^ exist that can be applied to the study of how salmon and sea lice interact. Less work has been put into repeating studies that quantify the level of sea lice that is lethal for a post-smolt. This is clearly a pivotal area of study that needs more attention. One method of collecting data on of infestation levels, proposed by Vollset *et al*. (2017), is experimental infestation of individual fish, tagging them (e.g. with passive integrated transponders) and releasing them into the wild. When these are then recaptured as adults the effect of varying degree of infestation levels can be assessed.

Even though sea lice was parameterized to affect the growth of the salmon, this had no effect on the ultimate size of the returning after one winter at sea. This occurred because reduction in size was also linked to decreased likelihood of survival. This could explain why the effect of sea lice on the size of returning salmon is generally small^23^. If the model predictions are correct, it is perhaps not surprising that we do not see a larger difference between treated and untreated fish when they return as adults. In contrast, the effect on age at maturation was strongly impacted by sea lice. This was mainly because the maturation equation takes into consideration the likelihood of survival and growth. Consequently, the effect on age at maturation is even stronger when sea lice were parameterized to affect the growth of the salmon. In this aspect, the model seems to deviate from the release groups studies. For example, Vollset, *et al*.^24^ demonstrated a significant, albeit relatively small, effect of treatment on proportion maturing after one year at sea. This could either reflect that the model exaggerates the effect of sea lice on age at maturation, or that the release group studies indeed reflect that these groups in general experience a generally low infestation pressure.

The impact of sea lice on the marine survival of salmon is one of the most debated topics in salmonid ecology, because of the complexity in acquiring unambiguous data to document the effect (if any). Marine survival of Atlantic salmon is variable and complex to study and most likely consists of several interacting factors acting together in time and space. This complexity is not uncommon when studying marine survival (or recruitment). However, when implementing management systems that link a management action (in this case keeping lice levels on fish farms low) to a specific survival of a target species, evidence-based decision making is crucial. In comparison with the risk evaluation suggested by Taranger, *et al*.^10^, and applied to modelled post-smolts in Kristoffersen, *et al*.^36^, the model predicts a larger effect when infestation pressure is low, while similar predictions (on average) are obtained when infestation pressure is high. Perhaps the biggest difference between this model and the risk evaluation of salmon used in various publications is that (1) the individuals are subject to size selective mortality during and after the process of encountering lice, and (2) that the host and parasite development and growth are affected by temperature. This simulation has yielded important results including previously undetected temperature-mediated risks associated with lice parasitism that could alter the balance between parasite and host in the wild.

## Conclusions

The model predicts a variety of patterns that can be tested: (1) Temperature mediated growth indicates that effects of sea lice should be stronger with warming water. This can have implications for the effect of parasite-induced mortality during the season, with latitudinal differences. (2) Initial size or growth can modulate the effect of sea lice-induced mortality. (3) Conditions that decrease the time spent in the nearshore areas (such as high river discharge or swimming speed) will reduce the encounter with sea lice.

## Supplementary information

Appendix
