# Supplementary material for: Parasite induced mortality is context dependent in Atlantic salmon: insights from an individual-based model
Source: Sci Rep. 2019 Nov 22;9:17377. doi: 10.1038/s41598-019-53871-2 (PMC6874588; doi:10.1038/s41598-019-53871-2)
Supplement: Supplementary file 1 — Appendix [file 41598_2019_53871_MOESM1_ESM.docx]

**Appendix**

**Parasite induced mortality is context dependent in Atlantic salmon: insights from an individual-based model**

**Vollset K.W.** ^1^*

^1^ NORCE Norwegian Research Centre, LFI - freshwater biology, Nygardsporten 112, 5006 Bergen, Norway

*Corresponding author, email: [knvo@norceresearch.no](mailto:knut.vollset@uni.no), tlf: 55584723

Running title: *Context dependent parasite mortality in salmon*

**Spatial infestation pressure**

Infestation pressure data was provided as raster files of modelled infestation pressure as described in Kristoffersen et al. (2017). The spatial pattern in infestation pressure is taken from a extracting the infestation pressure along the assumed migration route of the Voss salmon (black line – figure 1). For consistency, we use the infestation pressure modelled for week 22 in 2016. Week 22 is the week were most of the wild salmon in this region is found in the outer fjord system according to Vollset et al. (2016). The migration route is informed by the prevailing surface currents in the system based on a hydrodynamic model described in Barlaup (2013).


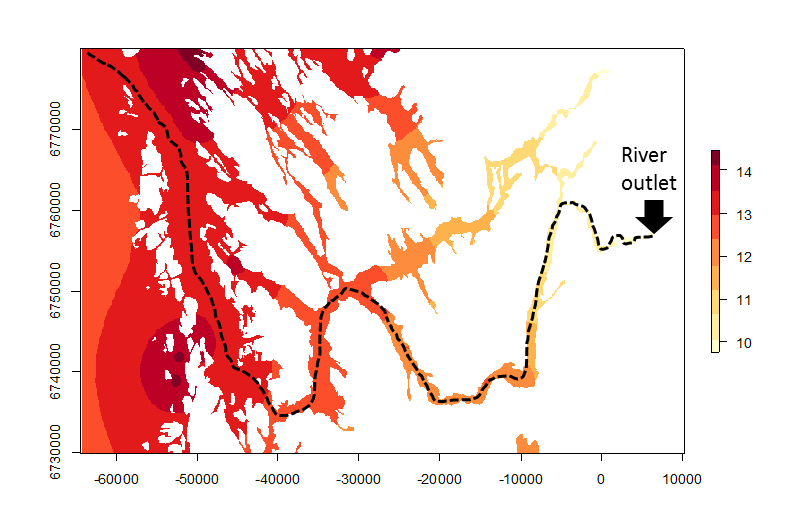


**Figure 1** Infestation pressure and assumed migration route of Vosso salmon smolt

The infestation pressure is extracted by using the function “extract” from the package *raster* with a buffer of 10. High and low infestation pressure is given in the model as the infestation pressure times a factor of 1.2 and 0.8 respectively. The resulting infestation pressure along the migration route for the three infestation pressures is given in Figure 2.

**Figure 2** Infestation pressure for low (dotted line), baseline (solid line) and high infestation pressure (dashed line) along the assumed migration route of the Vosso salmon.

Salmon lice encounter was modelled according to Kristoffersen et al. (2017), which used sentinel cages with salmon smolt to scale the infestation pressure from fish farms derived from Kristoffersen et al. (2014) to encounter rate with salmon lice. The relationship between IP and transmission pressure TP was accordingly

TP${=e}^{-14.063+0.843IP}$

The resulting sea lice encounters for the three infestation pressures

**Figure 3** Resulting number of lice encountered during migration for low, baseline and high infestation pressure

**Values used in sensitivity analysis**

Growth model

Growth was modelled using a temperature dependent model described in Handeland et al. (2003). The model is third order polynomial model with temperature (T) as the only explanatory variable

$$sgr=-0.000117+0.011395T+0.018436T^{2}-0.000977T^{3}$$

where *sgr* is the specific daily growth rate. To increase and decrease the growth rate the second parameter was multiplied by a factor of 2 and 0.2. This changed the growth rate by approximately 10% up or down throughout the thermal range.

Swimming model

Migration speed (or progression rate) was modelled using the model by Vollset et al. (2016). The modelled was simplified to only include size and discharge

$$km\times d^{-1}=-1.07+0.82\times L_{T}+0.017 \times m^{-3}s^{-1}$$

in which $km\times d^{-1}$is the daily migration rate of smolt, L_T_ is the length of the fish in mm, and $m^{-3}s^{-1}$ is the discharge from the river Vosso. To increase and decrease the migration speed the intercept was multiplied by a factor of 3 and 0.5. This changed the swimming speed by approximately 10% up or down throughout its range.

Salmon lice induced mortality

The likelihood of survival was fitted to lice per gram fish with a logistic regression. The data was extracted from the values used in Taranger et al (2012).

| Interval (interval) | Likelihood of survival (interval) |
| --- | --- |
| 0-0.1 | 1 |
| 0.1-0.2 | 0.8 |
| 0.2-0.3 | 0.5 |
| >0.3 | 0 |

These values are intervals and to be able to fit a logistic regression we had to interpret the interval as a single point. The following values were therefore selected

| Lice per gram fish (point) | Likelihood of survival | Max-values | Min-values |
| --- | --- | --- | --- |
| 0.1 | 0.9 | 0.8 | 0.999 |
| 0.2 | 0.8 | 0.5 | 0.9 |
| 0.3 | 0.5 | 0.2 | 0.8 |
| 0.7 | 0.001 | 0.001 | 0.001 |

The following fits were made to the point values in the table above

$Standard: log\left[ \frac{p}{1-p} \right]=4.272-15.70\times LPG$

$$Max:log\left[ \frac{p}{1-p} \right]=2.764-13.818\times LPG$$

$$Min: log\left[ \frac{p}{1-p} \right]=7.871-21.462\times LPG$$

where p is the likelihood of survival , and LPG is lice per gram fish. The values and fitted lines are plotted in figure A4


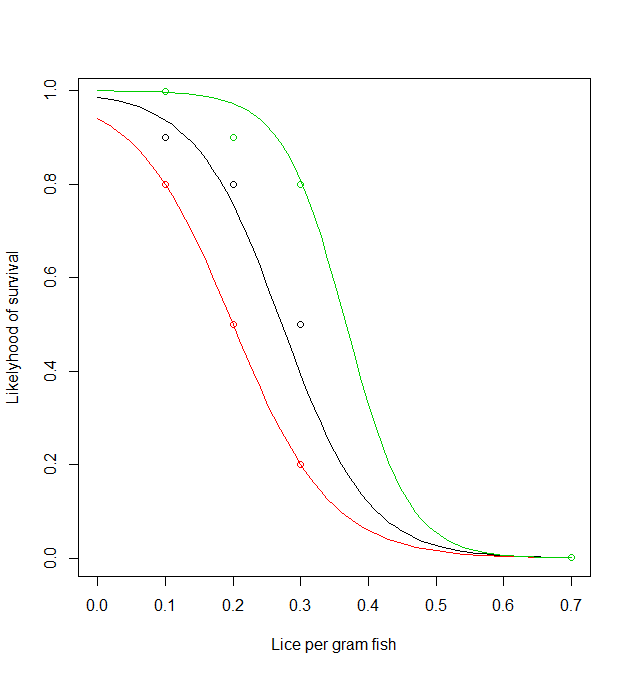


**Figure A4** Likelihood of survival according to lice per gram fish for baseline (black), high (green) and low salmon lice induced mortality

Encounter model

In the sensitivity, analysis the relationship between TP and IP (as explained above) was tested by varying the intercept with a value of ±2 which is approximately the variation observed in the random effects in the original model by Kristoffersen et al. (2017).

Mortality equation

Mortality was modelled using the size dependent model by Peterson and Wroblewski (1984)

$$Mp=5.3\times{10}^{-3}{({10}^{-3}DW)}^{-0.25}$$

Were Mp is the daily natural mortality and DW is the individual dry weight of the fish. DW is assumed to be 25% of the wet weight as logged in the model. To test whether a stronger or no size selectivity would affect the effect of salmon lice strong size selection was defined as follows

$$Mp=2.65\times{10}^{-3}{({10}^{-3}DW)}^{-0.5}$$

While no size selection was defined by setting Mp to 1. The three functions are plotted for the size range of 1:200 grams in figure

**Figure A5** likleyhood of survival per day according to mass for the baseline (solid line), strong (dashed line) and no size selective mortality.

List of abbreviations

M Mass of fish in gram

L_T_  Total length of fish

sgr Spesific growth rate

T Temperature

reduction Reduction in growth due to sea lice (%)

LPG Lice per gram fish weight


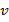
 Progression rate (velocity) of salmon smolts transiting through the fjord (km/day)

*D* Discharge from the Vosso river (m^3^s^-1^)

TP Transmission pressure as described in Kristoffersen et al. (2017)

IP The infestation pressure based on the estimated total external infestation pressure from fish farms on the Norwegian coast

theta the dispersion parameter in a negative binomial distribution used to model the patchiness of sea lice encounter

$\tau_{ij}$ minimum required development time for salmon lice *i* in stage *j* at a given temperature

$\beta$ parameters used to estimate $\tau_{ij}$described in Stien et al. (2015)

νj a stage‐specific constant (described in Groner et al. 2013)

Mp Daily natural mortality of salmon

p probability of sea lice induced mortality in logistic regression

log natural logarithm

m is the probability of surviving given an LPG

Ml Assumed daily sea lice induced mortality

DW Dry weight of fish

Sp Daily probability of survival

l_x_ the survival probability from birth to the beginning of the spawning season at age *x*

m_x_ the number of zygotes produced by an individual spawning at age *x*,

r population growth rate

F Fecundity

r1 estimate of *r* maturing after 1 year at sea

r2 estimate of *r* maturing after 2 year at sea

SW Sea winter

**References**

Barlaup, B.T. 2013. Redningsaksjonen for Vossolaksen. DN-utredning.

Kristoffersen, A.B., Jimenez, D., Viljugrein, H., Grontvedt, R., Stien, A., and Jansen, P.A. 2014. Large scale modelling of salmon lice (Lepeophtheirus salmonis) infection pressure based on lice monitoring data from Norwegian salmonid farms. Epidemics-Neth **9**: 31-39.

Kristoffersen, A.B., Qviller, L., Helgesen, K.O., Vollset, K.W., Viljugrein, H., and Jansen, P.A. 2017. Quantitative risk assessment of salmon louse-induced mortality of seaward-migrating post-smolt Atlantic salmon. Epidemics-Neth.

Peterson, I., and Wroblewski, J. 1984. Mortality rate of fishes in the pelagic ecosystem. Can J Fish Aquat Sci **41**(7): 1117-1120.

Vollset, K., Barlaup, B., Mahlum, S., Bjørn, P., and Skilbrei, O. 2016. Estimating the temporal overlap between post-smolt migration of Atlantic salmon and salmon lice infestation pressure from fish farms. Aquacult Env Interac **8**: 511-525.
